# Criticism on a Recently Reported Case—Scarlatinal Sequela, Treated with Veratrum Viride

**Published:** 1871-04

**Authors:** H. W. Boyce

**Affiliations:** Geneva, Wis.


ARTICLE III.—*Criticism on a Recently Reported Case—Scarlatinal Sequela, treated with Veratum Viride.* By H. W. BOYCE, M.D., Geneva, Wis.

My attention has been called to "Cases in Practice," February number of CHICAGO MEDICAL JOURNAL, Art. 1, of which one, "Case 4," was under my care. The report of "Cases" to be worth anything to the profession, should set forth all the facts truthfully.

On reference to my books, I find that Ida G—— was attacked with *Scarlatina Anginosa*, on Nov. 24th, 1870. Nothing worthy of note occurred during the febrile attack. The fever declined, and desquamation of the cuticle commenced about the fifth day. Subsequently, while attending the father of Ida G——, my attention was called to a slight sub-cutaneous œdema, affecting the eye-lids and face of the little girl. Coincident with the dropsy, there was slight febrile movement, with a pallid countenance. As these symptoms pointed unmistakably to *renal* disease, the ordinary sequel of scarlatina, "acute desquamative nephritis," I prescribed saline cathartics, fomentations over the kidneys, and sudorifics.

But little attention was devoted to this patient, as these measures appeared to be producing a favorable effect and an abatement of the disease. Early on the morning of Dec. 12th, I was called to see Ida, the messenger stating that she was in a fit. I then learned that early in the evening of the night previous, she had been attacked with pain in the head, and vomiting, which had continued during the night. Before I reached the house, the parents had placed her in a warm bath. She soon came out of the convulsions. I then directed the attendants to apply ice to the head, sinapisms to the feet and ankles, and fomentations over the kidneys, with warmth to the lower extremities. As the stomach had become too irritable to retain medicines, I directed them to give enemas of Sul. Magnesia, every hour, the bowels not having been moved during the night. I hoped that these measures would be sufficient to ward off the danger threatening my little patient, (uræmic poisoning). In this I was disappointed. The convulsions returned with increased violence, and of a decidedly epileptiform character. I then gave Calomel, grs. 15, hoping that this, with the enemas, would produce free evacuations of the bowels. In this I was not disappointed; towards evening there were frequent movements of greenish fœcal matter. This, with hot air baths, and the application of warmth to the lower extremities, finally checked the convulsions. The case was evidently complicated with uræmic poison. Hence the treatment, as far as practicable, must be directed to the elimination of urea from the blood. This was attempted by restoring the function of the kidneys, the skin, and gastro intestinal canal.

To fulfil these indications, I prescribed:

℞. Calomel, gr. xij; Nitrate Potass. ʒj; Pul. Digitalis, gr. iv. M. ft. ch. No. xij.

One to be given every two hours. Fomentations of Spts. Terebinth, to be applied over the kidneys, and to have water to drink freely, with sinapisms to feet and ankles. The head to be kept cool by cloths wrung out of cold water or ice.

This comprised the treatment, with occasional enemas during the night, and until Dr. B. O. Reynolds was called in. On consultation, it was thought best by Dr. R. to give inhalations of Sul. Ether and Fl. Ext. Valerian; to substitute Chlo. Potass. for Sul. Magnesia, in the enemas, and give Decoct. Fol. Buchu, and Bac. Juniper. I concurred. She was extremely restless, with slight convulsive movements of the limbs, which it was hoped the inhalations would allay. The Chlo. Potass. enema produced such irritation that it was abandoned after the first trial. But little of the Decoct. Buchu and Juniper could be taken. The Sul. Magnesia was substituted for Chlo. Potass., and continued until evening.

On the second consultation with Dr. R., it was concluded to try the effect of Veratrum Viride, if an opportunity should occur, in place of the Sul. Ether and Tr. Valerian. As the Dr. was to spend the night with the patient, it was left with him to carry out the programme. No other alteration in the treatment was suggested. Early the next morning, the father of the patient called me up and said that the Dr. had given two doses of the medicine, (Veratrum) and thought it helped her; that he had gone home, and he wished me to see if any more should be given. I examined the patient, but failed to see any marked improvement. Pulse and respirations remained extremely rapid, and there was great restlessness. In truth, the case looked very unpromising. As the extremities were cool, I had the warmth re-applied, and concluded that if the Veratrum had been beneficial during the night, I would continue its use. Looking around for the vial I found it gone. Thinking that the Dr. had *inadvertently carried it home*, I went to his house (not more than half a block from the patient), called him up, and inquired for the vial of Veratrum. In conversation with him, the Dr. said that he had given two doses of five m. each, and thought it had a good effect, but was doubtful if more could be safely given. Procuring the vial I returned to the house and gave three drops, watching the pulse for its effect. I thought I could perceive a slight diminution in the number of beats, with less restlessness. During the day I gave the Veratrum in small doses in combination with Acet. Ammonia, whenever indicated by the restlessness. As the day advanced, the patient become more quiet, the urine increased in quantity, with frequent alvine evacuations. To these changes in the secretions, I attributed the favorable change in my patient. The Veratrum, as far as I could see, produced no other beneficial effect than that of a sedative and sudorific. If the patient took the Veratrum under his direction as stated in the JOURNAL, then she got rather more than a double dose, and I cannot help but be thankful, that we did not kill her outright.

Through the JOURNAL I first learned this fact, and consider it most extraordinary, as up to this I had supposed that the patient was under my care, and that I was responsible for her treatment as her regular medical attendant, and that the Dr. was advising me as to the treatment of the case. During the night, he had charge of her treatment, but with the morning I again took charge of the case, and kept it until the patient was convalescent as I supposed.

With the elimination of the urea from the blood, the restoration of the renal function—the patient convalesced without any further complications from the disturbance of the renal functions.

### REMARKS.

In the review of this case, I am impressed with the conviction that the Dr. made a mistake in the proper diagnosis. It was not I think, a pure case of uræmia *per se*, but that the uræmic poisoning was due to the defective elimination of urea by the kidneys, and that this was due to the diseased condition of the kidneys, familiar to practitioners, as the dropsical affection which is apt to occur as the sequel of scarlatina, and was the cause of its accumulation in the blood at this time. If this view of the case was correct, then a rational treatment would have to be directed to the use of such remedies as would aid the vital forces in eliminating this poison from the blood through the kidneys, the skin, and gastro intestinal canal, if the Dr. had not discovered a specific for uræmic poisoning in the use of Veratrum. This rendered other remedies unnecessary. Perhaps if I had bled this patient at the commencement of the convulsions, I should have saved myself much anxiety in the after treatment of the case.

What beneficial effect had the Veratrum if any, in this case? To me it was apparent that the benefit was derived from its sudorific property, and sedative effect upon the nervous system, thus economizing the vital force expended in the restless, spasmodic movements, without checking secretion. The mere fact of lessening the pulse by the use of Veratrum was of no importance, without at the same time eliminating urea from the blood, that being the cause of the unnatural action of the heart. As to the question, "Is it a poison?" it is too absurd to require comment. In conclusion, allow me to say, in general terms, that the vaunting of heroic remedies, as specifics and cure alls, savors of quackery, and is on a par with Prof. J. Walter Scott's cancer plant, and others of the same class. No application is intended.

